# Pan-RAS inhibitors and polo-like kinase 1: promising targets in colorectal cancer

**DOI:** 10.1038/s41388-025-03484-z

**Published:** 2025-07-04

**Authors:** Priya Jayachandran, Andrew Elliott, Shivani Soni, Francesca Battaglin, Pooja Mittal, Sandra Algaze, Jae Ho Lo, Yan Yang, Karam Ashouri, Evanthia T. Roussos Torres, Wu Zhang, Joshua Millstein, Lin Zhang, Jian Yu, Heinz-Josef Lenz

**Affiliations:** 1 Division of Medical Oncology, Norris Comprehensive Cancer Center, Keck School of Medicine, University of Southern California, Los Angeles, CA USA; 2 Caris Life Sciences, Phoenix, AZ USA; 3 Department of Population and Public Health Sciences, Keck School of Medicine, University of Southern California, Los Angeles, CA USA

**Keywords:** Cancer genomics, Colorectal cancer

## Abstract

*RAS* is an oncogene that is commonly mutated in colorectal cancer (CRC). It has been considered a negative feature both due to its impact on prognosis and due to the shallow interface of oncogenic Ras for therapeutic targeting. Newer pan-Ras inhibitor strategies include improved direct targeting of RAS, blockade of downstream effectors, immunotherapy approaches, and even the inclusion of anti-EGFR drugs. Polo-like Kinase 1 (PLK1) is a serine/threonine protein kinase that controls multiple aspects of the cell-cycle. It is upregulated in CRC and has become an important therapeutic target in *KRAS* mutant CRC, with several PLK1 inhibitors currently in various phases of development and testing. As with other targeted therapies, resistance remains a problem and combination strategies may be beneficial. This review discusses pan-RAS inhibitors and PLK1 in the context of CRC. It discusses *RAS*’ many roles, its associated pathways and relationship to cancer progression, the current status of existing inhibitors, and future strategies for targeting in cancer therapy. The wide-ranging impacts of *RAS* provide a basis to better understand and fight against CRC.

## Introduction

CRC is one of the most common cancers in the world. Despite advances, long-term survival remains disappointing for patients with metastatic disease (mCRC) [1]. Over the last few decades, outcomes have improved through combination chemotherapy and the introduction of targeted therapy [2, 3]. Mutations in many genes, including *RAS* family genes*, PIK3CA, BRAF*, and *PTEN*, can influence cell proliferation in CRC, as well as p53, TGF-β, and MAPK/PI3K pathways [4].

There are three *RAS* oncogenes—Kirsten rat sarcoma virus (*KRAS*), *HRAS*, and *NRAS* – located on the short arm of chromosome 12 [5]. *RAS* is frequently mutated in CRC and other cancers [5]. Around 40% of CRCs harbor *KRAS* mutations, with around 65% of those in mCRC harboring G12 glycine hotspot mutations [6, 7]. G12C mutant cancers generally have lower response rate to chemotherapy and worse prognosis. Epidermal growth factor (EGFR) blockade can lead to acquired resistance via *RAS* mutations [8].

**Fig. 1:**
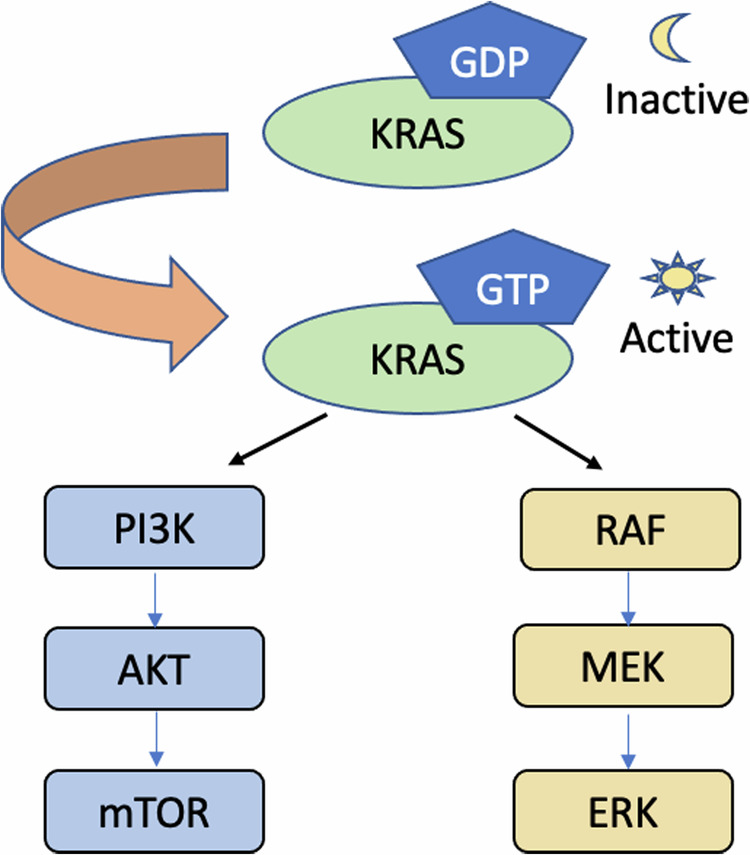
Overview of Ras pathways.

RAS proteins are guanosine triphosphate (GTP) phosphatases (GTPases) that regulate many pathways vital to cell proliferation, migration, and differentiation [9]. KRAS proteins are inactive when GDP-bound and active when GTP-bound. Activated RAS triggers multiple downstream pathways, such as mitogen-activated protein kinase (MAPK; RAS-RAF-MEK-ERK) and phosphatidylinositol 3-kinase (PI3K; PI3K-AKT-mTOR) [10] [Fig. 1]. By doing so, oncogenic *RAS* mutations promote cell proliferation and cancer growth.

Oncogenic *KRAS* mutations are associated with worse outcomes and resistance to many treatments [6, 11]. This is especially true in microsatellite stable (MSS) disease [1] with *RAS* mutations where anti- EGFR therapies, such as panitumumab and cetuximab, are ineffective [12–14], and the standard of care is chemotherapy with anti-vascular endothelial growth factor therapy [15]. Despite many recent advances, *KRAS* mutations still represent an important area of unmet need [16–18]. Mutant *KRAS* (*KRAS*-MT) cells are particularly dependent on mitotic function genes such as *PLK1*, which will be discussed further.

## RAS-targeting therapies

### Direct binding RAS inhibitors

The structure of RAS limits the development of direct-binding RAS inhibitors due to its overall small size and shallow binding pocket. SCH-53239, which directly competes with GDP binding, was one of the first inhibitors developed [19]. However, it was never approved by the Food and Drug Administration (FDA) due to toxicity and the lack of a clearly distinguished binding pocket [19]. GTP is present in high concentrations intracellularly and binds to KRAS with high affinity, making GTP competitive inhibitors challenging to pursue. Other inhibitors, such as KAL-21404358, bind to different sites, such as on KRAS^G12D^ to impair the interaction with BRAF and disrupt RAF-MEK-ERK and PI3K-AKT signaling pathways [20]. BI-2852 binds to both active and inactive KRAS to inhibit downstream signaling and blocks proliferation of *KRAS* mutant cells [21]. Better understanding of KRAS structure and the Switch binding pocket region have broadened the horizons for direct targeting, especially with KRAS^G12C^.

### KRAS G12C mutation

*KRAS*^*G12C*^ has recently dominated the field as a novel druggable target [22]. This mutation occurs in 13% of non-small cell lung cancer (NSCLC) but only in 1–3% of CRC and other solid tumors. The G12C mutation is a glycine to cysteine substitution at codon 12 that favors the activated state of *KRAS* and amplifies oncogenic signaling pathways. Direct inhibitors are essentially able to freeze KRAS^G12C^ in its inactive state [23]. ARS-853, one of the first KRAS inhibitors targeting G12C mutations, was able to bind to the Cysteine 12 and Switch II pocket region of the inactive oncoprotein, to successfully induce apoptosis and inhibited MAPK and PI3K signaling [24]. However, its translation into clinical use was unsuccessful due to its short half-life. Sotorasib was the first clinically used KRAS^G12C^ selective inhibitor. It was FDA approved in 2021 for use in NSCLC based on the CodeBreaK 100 single-arm, phase 2 clinical trial [25]. In a phase 3 trial, sotorasib demonstrated superior progression-free survival PFS versus docetaxel in patients with advanced *KRAS*^G12C^ mutant NSCLC [26]. Less frequent response was seen in CRC with a 7.1% overall response rate (ORR) [27]. The differential response may be, at least in part, due to the more variable molecular heterogeneity of mCRC compared to NSCLC.

Adagrasib is another *KRAS*^*G12C*^ inhibitor which was tested in the phase 1b/2 KRYSTAL-1 trial and granted FDA approval in 2022 for NSCLC [28]. The combination of adagrasib plus cetuximab showed promising results in heavily pretreated patients with metastatic *KRAS*^G12C^ mutant CRC with an ORR of 34.0% and disease control rate (DCR) of 85.1% [29]. Median PFS was 6.9 months. In June 2024, the FDA granted accelerated approval to the combination, which has become a standard of care option per NCCN guidelines (sotorasib or adagrasib + cetuximab or panitumumab as biomarker directed therapy) [29]. The phase 3 CodeBreaK 300 trial evaluated the combination of sotorasib with anti-EGFR for chemorefractory metastatic CRC with mutated *KRAS* G12C. The median PFS was 5.6 months in the 960-mg sotorasib–panitumumab group compared with 2.2 months in the standard-care group [30]. Sotorasib has also been combined with various agents of different classes [31]. Sotorasib induced an inflamed tumor microenvironment with increased T-cell activation and combination treatment with sotorasib and anti-PD-1 improved response in mouse models [32]. Several phase 1/2 trials are ongoing, combining anti-*KRAS*^*G12C*^ agents with inhibitors of other classes (including immune checkpoint, MEK, SHP2, pan-HER, EGFR) and cytotoxic agents [33]. Optimal second-line treatment of *KRAS*^*G12C*^ mCRC is being explored in the phase 3 KRYSTAL-10 study, randomizing to either adagrasib and cetuximab or chemotherapy (NCT04793958). Another agent, divarasib, is also being studied in combination with cetuximab in a phase 1b trial.

### Non-G12C KRAS mutations and anti-EGFR

Although recent focus has centered on the G12C mutation, other G12 mutations have also been targeted, especially in combination with anti-EGFR agents. A global collaborative database called RASCAL (the KRAS in colorectal cancer collaborative group) collected information on tumor genotype and outcome in CRC. Only G12V mutations demonstrated significantly inferior prognosis in stage III CRC and regardless of treatment [34]. Cetuximab (when combined with chemotherapy) may confer greater benefit to patients with *KRAS*^G13D^ tumors versus other mutations [35].

However, in a recent pooled analysis of multiple studies, no *KRAS* mutant allele was shown to be a predictive biomarker for response to panitumumab [36]. The phase 2 ICECREAM trial assessed cetuximab compared to cetuximab + irinotecan in *KRAS*^G13D^ mutated chemorefractory mCRC patients and suggested a benefit to cetuximab treatment [37]. While *RAS* mutations are known to cause resistance to anti-EGFR therapies in mCRC, different codon mutations may lead to differential responses. *KRAS*^G12D^ has specifically been targeted given its prevalence in CRC. MRTX1133 is a *KRAS*^G12D^ inhibitor that has recently shown efficacy in vitro and in PDX models [38]. There is some evidence that the drug may modulate the immune microenvironment and testing is now moving into human phase 1/2 trials [38].

Afatinib and neratinib are anti-HER inhibitors. They showed efficacy in preclinical models of *RAS* mutant mCRC, but clinically, the response was quite limited (12%) [39]. Imgatuzumab, an engineered anti-EGFR mAb, showed limited efficacy in *KRAS* mutant mCRC, either alone or with FOLFIRI chemotherapy [40]. Truly pan-*RAS* broad inhibitors have been considered, although toxicity would likely be a major concern with this approach [9].

Rigosertib is a multi-target inhibitor that was initially thought to effect PLK1 and later various aspects of RAS signaling as a microtubule destabilizer. It did not show any benefit in a clinical trial [41]. However, other compounds have demonstrated activity against specific mutations, including *KRAS*^*G12D*^ and *KRAS*^*G12V*^, in CRC and other malignancies. The *KRAS*^*G12D*^ mutation is the most common *RAS* mutation in colon cancer, and thus, a specific G12D inhibitor might be the most helpful in this setting [42]. Mirati Therapeutics is investigating agents targeting *KRAS*^*G12D*^ in phase 1 and 2 studies. Revolution Medicines confirmed plans in 2024 for two phase 3 trials of its multi-KRAS inhibitor RMC-6236 [43]. RMC-6236 showed potential in NSCLC and pancreatic cancers with various non-G12C *KRAS* mutations. The phase 1 study reported response rates of 38% and 20% in lung and pancreas, respectively, but with caveats of an unclear dose-response pattern and concerns about rash toxicity [43]. Another oral pan-RAS inhibitor known as RMC-7977, with high selectivity for GTP-bound *KRAS*, *HRAS*, and *NRAS* (regardless of mutational status), effectively targets the common cancer-causing RAS proteins while minimally impacting normal cells [44].

### SOS1 and SHP2 inhibitors

SOS1 is as a guanine nucleotide exchange factor (GEF) that helps activate KRAS. Blocking SOS1 directly inhibits KRAS and impairs the survival of *RAS*-mutant CRC cells [45]. A phase 1 trial is evaluating the SOS1 inhibitor BI-170196 both as monotherapy and in combination with the MEK inhibitor trametinib (NCT04111458). BAY-293, another small-molecule inhibitor shows synergism with a KRAS^G12C^ inhibitor by increasing available inactive GDP-bound KRAS^G12C^ [46]. A very recent study of the SOS1 inhibitor BI-3406 plus adagrasib in lung and CRC models showed improved responses with the combination and also delayed emergence of acquired resistance [47].

SHP2 is a protein that increases SOS1 activity [46]. It is a protein tyrosine phosphatase essential for GTP binding on *RAS* and activation of the MAPK pathway [48]. One selective SHP2 allosteric inhibitor was found to decrease cell proliferation in early studies with best response seen in *KRAS*^G12C^-mutant cells [48]. Inhibitors of SHP2 (alone or in combination with MEK inhibitors) are also in phase 1/2 clinical trials. A SHP2 inhibitor TNO155 combined with a KRAS^G12C^ inhibitor JDQ433 showed efficacy in patients with *KRAS*^*G12C*^ mutant cancers [49]. The combination is thought to move KRAS to the inactive state and also inhibit reactivation.

### Targeting MAPK and signaling pathways

Oncogenic RAS activates both the RAS/RAF/MEK/ERK and PI3K/AKT/mTOR pathways, which are essential to the growth and proliferation of cancer [50]. Inhibiting one pathway may lead to increased signaling of the other, so combining therapeutic agents to block both pathways could be an effective strategy to stymie the downstream pathways of RAS signaling.

### MEK inhibitors

MEK inhibitors (MEKi) represent another approach in treating *RAS* mutant mCRC. MEKi block phosphorylation of ERK1/2, preventing its nuclear translocation and downstream MAPK pathway effects [51]. Trametinib and cobimetinib alone were not active in CRC patients [52]. This may be due to redundant signaling through upstream receptor tyrosine kinases and parallel pathways. ERK inhibitor monotherapy also did not demonstrate significant response (NCT02857270, NCT02313012). Given the disappointing results as single agents, combinations approaches have tried to block these redundant signaling pathways.

Selumetinib (AZD6244) is an oral MEK 1/2 inhibitor [53]. Selumetinib turns MEK1/2 into their inactive conformations so they are unable to activate ERK. Selumetinib was compared to capecitabine in a phase 2 study of oxaliplatin and irinotecan refractory CRC and demonstrated reasonable tolerability and similar efficacy, but no objective response [54]. Combining inhibition of RAF and MEK together may be another approach to exploit.

MEKi and anti-EGFR drugs have been combined in an attempt to circumvent anti-EGFR resistance in *RAS*-mutant mCRC [55]. MEKi combined with cetuximab, lapatinib (anti-HER2), or the EGFR/HER3 dual inhibitor duligotuzumab yielded some stability at best, but unfortunately no tumor regression or true responses [56]. Likewise, the combination of PIK3CA inhibitor copanlisib with refametinib was not found to have a tolerable and efficacious dose schedule [57]. Binimetinib with buparlisib was also tested [58]. Phase 1 trials combining MEKi with neratinib, crizotinib, or panitumumab are underway. Combinations with CDK 4/6 inhibitors have also been tested [59].

### CDK 4/6 inhibitors

MAPK pathway activation can contribute to G1-S phase progression through the cyclin-dependent kinase (CDK) pathway [59]. Single agent palbociclib yielded only a 0% ORR and 33% DCR in a phase 2 trial [60]. However, trametinib combined with palbociclib showed efficacy in *KRAS* mutant CRC xenograft models and pancreatic cell lines [61, 62]. Lee et al. found that MEK and CDK4/6 inhibitors (binimetinib with palbociclib) worked well together to cause tumor shrinkage in vivo in preclinical models of *KRAS* mutant CRC [59]. This combination was tested in a phase 1b study showing preliminary safety data. However, a phase 2 trial of binimetinib plus palbociclib versus trifluridine/tipiracil (TAS-102) unfortunately did not show significant improvement in PFS in refractory *KRAS* mutant CRC [63].

### mTOR inhibitors

As with CDK4/6 inhibitors, single agent mTOR inhibitors appear to have minimal antitumor activity in *KRAS* MT mCRC despite their efficacy in breast cancer. Only 38% of patients had disease stability after treatment with temsirolimus in one study [64]. *PIK3CA* mutations can induce downstream mTOR signaling, which can in turn render MEKi ineffective [22]. Thus MEKi with PI3K or mTOR inhibitors are being explored and tested in mCRC, although several doublets did not work very well in practice. Disappointingly, no response was seen with any of the PI3K/mTOR dual inhibitors (gedatolisib, voxtalisib, or omipalisib) plus MEKi [57, 58, 65]. In a phase 1 study, the combination of a PI3K inhibitor and a MAPK inhibitor resulted in extremely variable tumor responses (2–64%) [66]. A recent cell culture study found that treatment with an MTOR inhibitor (Torin 1), AKT inhibitor (MK2206), and MEKi (selumetinib) lead to dramatic apoptotic cell death [67]. Additional early phase studies in cell lines and xenograft models continue to show the possible synergistic effect of MEKi and PI3K/mTOR inhibitors [68, 69].

RAS depends on a small GTPase called RAC1, which then activates p21-activated kinases (PAKs). PAKs control multiple processes essential to cancer growth including cell division and migration. Inhibition of PAKs thus halts proliferation of *KRAS* mutant CRC cells. However, prolonged blockade results in hyperactivation of the mTOR (p70S6K) pathway results. In one study, the addition of everolimus resensitized cells to a PAK inhibitor and controlled the growth of *KRAS* mutant CRC tumors, suggesting another promising therapeutic strategy [70].

### WEE1 and TRAIL receptor

WEE1 is a Ser/Thr protein kinase that regulates the cell cycle. Adavosertib, a small molecule inhibitor of WEE1 kinase, is a promising agent in *RAS*/P53 mutant mCRC that has been shown to be effective as monotherapy maintenance following induction chemotherapy [71]. Tumor necrosis factor (TNF)-related apoptosis-inducing ligand (TRAIL) specifically targets cancer cells while sparing normal cells, making it a desirable target to avoid many of the off-target side effects that are common with chemotherapy [72]. Eftozanermin binds to pro-apoptotic TRAIL death receptors. Resistance remains a major issue, but modulating the expression of TRAIL decoy receptors may hold therapeutic promise [72].

### Immunotherapy

Immunotherapy has emerged as a new standard first-line treatment for microsatellite instability-high (MSI-high) mCRC patients [18]. However, for patients with *RAS* mutations, pembrolizumab is not superior to cytotoxic chemotherapy. The majority of patients with mCRC attain little benefit because of the low immunogenicity of their cancer. *KRAS* mutations manipulate the tumor microenvironment (TME). Compared to *KRAS* wild-type tumors, the TME of *KRAS*-mutant CRC has fewer B cells, M1 macrophages, CD4 T cells, and neutrophils, but more Treg cells [73]. *KRAS* mutations stimulate an influx of cytokines to maintain an immunosuppressive TME, which decreases the efficacy of immune checkpoint inhibitors (ICI), especially alone. Multiple immune-related pathways, including interferon-γ (IFN-γ), are down-regulated in *KRAS*-mutant CRC [74]. It has been suggested that *KRAS* mutations might facilitate cancer immune escape mechanisms [75].

Recent studies suggest that treatment with RAS inhibitors, including *KRAS*^G12C^ inhibitors, SHP2 inhibitors, or MEK inhibitors, could trigger immune cells to effectively lift the immunosuppressive status and allow for ICI response in preclinical models of *KRAS*-mutant CRC [32, 76]. This supports the concept of combining ICI with *KRAS*-targeted therapies, but further clinical trial validation is still needed. The combination of sotorasib with ICIs boosted immune activity and tumor regression in 90% of PDX models derived from human *KRAS*^*G12C*^ mutant CRC cells [32]. The combination of adagrasib and anti-PD-1 agents has shown similar success [77]. The CodeBreaK101 and KRYSTAL-1 trials are evaluating sotorasib and adagrasib with anti-PD-1. However, a phase 1b/2 trial of nivolumab and binimetinib with or without ipilimumab in patients with *KRAS* mutant MSS mCRC recently showed a lack of response with either drug combination [78].

### Vaccines and adoptive cell therapy

KRAS-derived neoantigens can be perceived as “nonself” by T-cells, making them a therapeutic target. Cancer vaccines made with mutant RAS peptides have been demonstrated to trigger appropriate immune reactions in the majority of CRC patients [79]. The GI-4000 recombinant saccharomyces vaccine expresses mutant RAS protein and induced remission in preclinical models with a favorable safety profile in a phase 1 clinical trial [80]. Cancer vaccine ELI-002 2 P was designed to enhance delivery to the lymph nodes and immune response [81]. Twenty-five patients with minimal residual disease-positive KRAS mutant pancreatic or colon cancer were evaluated in a phase 1 study. Eighty four percent of patients demonstrated mutant *KRAS*-specific T cell responses and biomarker responses. The vaccine appeared safe and effective in patients with immunotherapy-resistant *KRAS* mutant tumors [81]. mRNA vaccines encoding novel KRAS mutation epitopes are being evaluated as monotherapy and with pembrolizumab. Pelareorep is an oncolytic RNA virus that selectively lyses *KRAS*-mutated CRC cells [82]. It induced 50% partial response when combined with FOLFIRI and Bevacizumab [83].

Based on stem cell transplant concepts, adoptive cell therapy (ACT) uses externally expanded tumor-reactive T-cells and administers them to pretreated patients [84]. With tumor infiltrating lymphocytes (TILs) therapy, the cells are taken from the TME within tumors. In T-cell receptor (TCR) therapy, peripheral blood lymphocytes are isolated and genetically engineered to express TCR targeting specific tumor antigens. A retroviral vector encoding a murine TCR is transfected into a patient’s peripheral blood lymphocytes, which are then expanded in the lab and reinfused into the patient to trigger an immune response to eliminate the cancer cells. KRAS mutant antigens presented by human lymphocyte antigen (HLA) can be recognized by the TCRs. A CD8 + T-cell response against mutant *KRAS*^G12D^ resulted in a dramatic shrinkage of lung metastases in a patient with mCRC using this approach [85]. Similar response was seen in a patient with metastatic pancreatic cancer and the modified T cells persisted in the circulation 6 months later. Further trials are studying T cells with anti-*KRAS*^*G12D*^ or anti-*KRAS*^*G12V*^ TCR. Lu et al. identified two TCRs specific for the *KRAS*^*G12V*^ mutant neoantigen which lead to cytokine release and cell death in culture [86]. T cell-mediated therapy is a novel approach that could overcome some of the challenges associated with both specific inhibitors and more generic immune treatments.

### Manipulating cellular metabolism

Cancer may reprogram core cellular pathways to provide energy for tumor growth. Amino acids are essential building blocks for this. *KRAS* mutations can further induce metabolic reprogramming [87]. KRAS regulates amino acid and fatty acid metabolism to promote cancer cell division and growth [88]. *KRAS* mutations upregulate amino acid transporters that bring amino acids into the cell. The transporter SLC7A5 is upregulated by KRAS and has effects on the transcription of multiple pathways, including MTOR [89]. Deletion of the transporter sensitized *KRAS*-mutant cancers to MTORi, such as everolimus. Telaglenastat is a glutaminase inhibitor that has been investigated in combination with palbociclib in a phase 1b/2 trial in pretreated *KRAS* mutant mCRC (NCT03965845).

GLUT1 is upregulated in *KRAS* mutant cancer cells. The glucose transporter allows cells to survive under hypoglycemic conditions by increasing glucose uptake and glycolysis. Inhibitors of glucose metabolism, including HIF1α inhibitor (IDF-11774) and GLUT1 inhibitor (WZB117), have shown promise in cell-line studies of KRAS-mutant CRC [90]. Fatty acid synthase (FASN) is a lipid synthesis enzyme that is frequently upregulated in *KRAS* mutant cells. A FASN inhibitor was explored in resectable solid tumors in a phase 1 trial (NCT02980029). Vitamin C, especially at high doses, has been a controversial therapy in oncology. It is true that *KRAS* mutant cells die when exposed to high doses of ascorbic acid (vitamin C) due to oxidative stress and oxygen radicals [91, 92]. The selective obstruction of the MAPK pathway by vitamin C in *KRAS* mutant CRC cells is also an intriguing factor to consider therapeutically. A phase 3 randomized trial found that combining high-dose vitamin C with chemotherapy did not improve PFS in mCRC but could still be helpful for those with *RAS* mutations [93].

### Statins

Statins are medications commonly prescribed to lower lipid levels. They could also play a role in treating *RAS* mutant CRC. Recently, they have been suggested to improve mortality and prognosis in several cancers. Statins block the mevalonate pathway, which disrupts the post-translational modification of the RAS protein [94]. They may induce apoptosis through downstream effects on ERK, AKT, and mTOR. Statins also interfere with RAS activation by blocking binding to the plasma membrane. Unfortunately, combining simvastatin did not seem to sensitize cancer cells to anti-EGFR drugs in clinical trials [94, 95]. A retrospective analysis with chemotherapy, bevacizumab, and cetuximab yielded negative results [96]. However, a recent study by Tsubaki et al. found that statins enhance the antitumor effect of oxaliplatin in KRAS mutant CRC cells and actually prevent oxaliplatin-induced neuropathy [97]. Another phase 2 trial showed a relatively impressive response to irinotecan, cetuximab, and simvastatin in irinotecan-refractory *KRAS* mutant mCRC with a 65.4% DCR and PFS of 7.6 months [98].

### Herbal and natural supplements

Herbs and spices contain many bioactive compounds which may be exploited for the anti-cancer effects. Some herbal compounds possess preventative and adjuvant effects in *KRAS* mutant CRC. Ginger extract inhibited cell growth in a CRC cell line (HCT-116) by downregulating *KRAS* and *MMP-2* gene expression. There are indications that turmeric, ginger, sesame, flaxseed, and fenugreek all have beneficial effects via BCL-2, KRAS, and MMP pathways and effects on oxidative stress and inflammation [99]. Some polyphenols, such as reservatrol found in grapes, may also be effective in treating *KRAS* mutant CRC. Via inhibition of PDE4 (phosphodiesterase-4) activity, resveratrol can inhibit the proliferation of *KRAS* mutant CRC cells [100]. Combining resveratrol with ferulic acid, an antioxidant found in plant cells, inhibits this proliferation even more effectively [100]. Quercetin is a flavonoid found in various fruits and vegetables. It induces apoptosis in *KRAS* mutant CRC cells through effects on the JNK and AKT pathways [101]. Luteolin and ursoic acid, compounds that are found in various fruits and vegetables, have also been shown to inhibit *KRAS* mutant CRC cell proliferation [102]. Curcumin, derived from turmeric extract of the rhizome of the Curcuma longa plant, is a MEK inhibitor. When combined with regorafenib, it has been shown to have a toxic effect on *KRAS* mutant CRC cells [103].

### AI/machine learning

Artificial intelligence (AI) is a field of computer technology that utilizes statistical methods to analyze large data and make predictions to simulate human problem solving and decision making. In recent years, AI has been applied to oncology to find novel approaches in the management of various cancers, including CRC. A machine-learning approach can be used to understand and exploit oncogenic pathways in tumors. One algorithm utilizes data from The Cancer Genome Atlas (TCGA), including RNA-seq, copy number alterations, and mutations [104]. The method can detect *RAS* activation and predict responses to various inhibitors. Increased RAS activity is noted with multiple aberrant states or hits in the pathway. While the transcriptome is currently underused in oncology, machine learning can be utilized to identify targets and predict responses. AI has already been used to evaluate MSI, EGFR, immunotherapy markers and other aspects critical to the best treatment of CRC. Modeling has been used as a high-throughput screening tool to identify potential *KRAS*^G12C^ inhibitors and inform drug design [105]. Recently, a deep neural framework has been proposed for “deciphering and identifying pan-cancer Ras pathway activation” (DIPRAS). DIPRAS gives insights from a deeper perspective, allowing for identification and characterization of aberrant RAS activity through gene enrichment and pathological analysis [106].

### PLK1

**Fig. 2:**
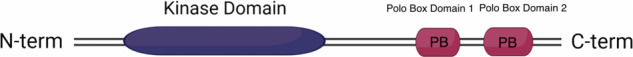
The PLK1 structure includes two functional polo-box domains (PBDs) at the C-terminal and the kinase domain at the N-terminal.

Polo-like Kinase 1 (PLK1) is a serine/threonine protein kinase that has emerged as a next generation target in the treatment of cancer [107]. It is coming to play a major role especially in the treatment of *KRAS* mutant CRC. It contains a kinase domain at the N terminus and two polo-box domains (PDBs) at the C terminus [Fig. 2]. As a cell cycle regulator, it is responsible for the G2-M transition and mitotic entrance [107]. PLK1 is also critical to cytokinesis and establishing centromeres, with knock-down leading to failed spindle assembly [108]. Plk1 homozygous null mice die at the eight-cell embryo stage. Plk1 heterozygotes are healthy at birth but have threefold greater incidence of tumors than their wild-type counterparts. PLK1 overexpression has been shown to trigger oncogenic transformation via epithelial-to-mesenchymal transition (EMT) and increased cell migration and invasion [109]. It strongly activates MAPK signaling [109].

PLK1 helps inhibit cell death pathways such as apoptosis. There is an association between high PLK1 and low immune cell infiltration and antitumor activity in many cancer types [110, 111]. PLK1 inhibits NF-κB transcription, which triggers cytokines essential to inflammation and immunity [112]. PLK1 is highly expressed in many human cancer types, including colorectal, breast, esophageal and gastric, endometrial, head and neck, and lung cancer [113]. Overexpression plays a role in the progression of colorectal cancer and inversely correlates with both disease free and overall survival [113]. Blocking the expression of PLK1 can inhibit the proliferation of tumor cells and induce apoptosis. Thus, multiple PLK inhibitors have been developed and evaluated in preclinical and clinical trials [113] [Table 1]. Initial studies of BI2536 were disappointing [114]. However, BI6727 (volasertib) was granted breakthrough therapy designation by the FDA in 2013 [115]. Onvansertib is a third generation ATP-competitive PLK1 inhibitor that was granted orphan drug designation by the FDA based on its efficacy in AML [116].

**Table 1:** Selected PLK1 inhibitors that are in various stages of development and trials.

Inhibitor	Company/Status	Class
BI2536	Boehringer Ingelheim—phase 2, discontinued	ATP-competitive
Volasertib (BI6727)	Boehringer Ingelheim—phase 3	ATP-competitive
TAK960 hydrochloride	Takeda Pharmaceutical Company—phase 1	ATP-competitive
Onvasertib (NMS-1286937)	Nerviano Medical Science/Cardiff Oncology—phase 2	ATP-competitive
Poloxin	Max Planck Institute of Biochemistry	Non–*ATP*-competitive
Rigosertib	Onconova Therapeutics, Inc.—phase 3	Non-ATP-competitive

Han et al. found that PLK1 was expressed in 73.2% of colorectal cancers and 3.6% of normal tissues and was associated with tumor size, extent of invasion, and lymph node metastases [117]. Treatment with siRNA oligos inhibited PLK1 in SW1116 cells with halting of cell proliferation followed by apoptosis after transfection. They suggested that PLK1 could be a useful biomarker for CRC patients given its association with PCNA (proliferating cell nuclear antigen). They also suggested that PLK1 suppression can inhibit colon cancer cell migration and invasion [117].

### PLK1 inhibitors

There are two main types of PLK1 inhibitors: (1) ATP competitors, which target the kinase domain; and (2) non-ATP competitors, which target the PBD domain [112]. ATP competitors include drugs such as volasertib or onvansertib. They have already been tested in phase 1–3 clinical trials for solid tumors as well as hematological malignancies with varying degrees of response [118, 119]. The results of preclinical studies, however, have not always panned out in clinical trials. For example, in preclinical studies, BI2536 inhibited the proliferation of various cell lines by blocking metaphase cell division leading to apoptosis. However, it was less effective in clinical trials, especially as a single agent [114, 120]. In fact, in one study there was almost no response in patients with advanced solid tumors [121]. In a Phase 2 study of patients with relapsed small-cell lung cancer, there was again no response and all progressed [122]. The disappointing results did not encourage further clinical investigation. Simvastatin was able to re-sensitize BI2536 resistant cells by targeting the mevalonate pathway and may be one strategy to manage PLK1 resistance [123].

Despite this, PLK1 inhibitors have shown efficacy in several clinical trials, at least stabilizing the disease or in some cases drawing partial responses. Volasertib has shown efficacy in vitro and in vivo and more importantly in phase 1 and 2 trials. Response seemed to vary by tumor type and the drug was explored further in the hematologic realm [115]. It showed excess toxicity in a phase 3 trial in acute myeloid leukemia [124]. Combinations with other inhibitors seemed encouraging prior to discontinuation [125]. As of 2024, there are plans to resurrect the drug in a phase 2 study.

Unfortunately, ATP competitive inhibitors are prone to resistance, non-specific effects on other kinases, and dose limiting toxicities [9]. Resistance commonly occurs due to mutations at the ATP-binding pocket, prompting further research to improve drug selectivity and efficacy by targeting the kinase domain residues unique to PLK1 [126]. The PBD, on the other hand, is far more specific and a promising target for drug development. Poloxin, a synthetic derivative of thymoquinone, which is derived from black cumin seeds, was the first small molecule inhibitor targeting PLK1 PBD to cause mitotic arrest and apoptosis [127]. Purpurogallin is another selective inhibitor that has been tested in vitro and in vivo [128].

Onvansertib is an oral, ATP-competitive PLK1 inhibitor that displays synergism with both irinotecan and 5FU in xenograft models [116]. *KRAS* mutant tumor cells show higher sensitivity to onvansertib compared with *KRAS* wildtype [129]. Kopetz et al. showed the combination of onvansertib and irinotecan significantly improved response versus single agent onvansertib, supporting that onvansertib can overcome irinotecan-resistance in *RAS*-mutated CRC [116]. Patients enrolled with a history of prior irinotecan treatment showed clinical benefit following onvansertib + FOLFIRI/bevacizumab treatment, particularly with early ctDNA decreases.

Lenz et al. first presented safety, efficacy, and biomarker data from a phase 1b trial of onvansertib with FOLFIRI-bevacizumab in patients with *KRAS* mutant mCRC after treatment with 5FU and oxaliplatin, ± bevacizumab [130]. Treatment was well tolerated with manageable side effects and early ctDNA changes were observed. In phase 2, the median DOR was 11.7 months, but ORR was as high as 76.9% and median PFS 14.9 months in patients who had not previously received bevacizumab [131]. Prior bevacizumab exposure led to onvansertib resistance, but combining the agents was effective via inhibition of hypoxia and angiogenesis pathways. The combination is thus very promising in the second-line treatment of *KRAS* mutant mCRC and now being evaluated in the first-line.

### PLK1, chemotherapy resistance, and immunotherapy combinations

PLK 1 has been found to inactive the p53 tumor suppressor gene consistent with the previously discussed synergistic effects of PLK1 inhibitors with irinotecan [132]. These p53 effects have also been associated with resistance to doxorubicin [110]. PLK1 also causes gemcitabine resistance in pancreatic cancer by driving DNA replication via HBO1 and ORC2 [133]. PLK1 phosphorylates substrates regulating microtubule dynamics and affects chromosomal instability resulting in paclitaxel resistance [134, 135]. Yu et al. found that the inhibition of PLK1-cell cycle control actually overcomes resistance to oxaliplatin in CRC [136]. PLK1 leads to HIF-2-dependent promoter targeting and VEGFA angiogenesis leading to sunitinib resistance in renal cell carcinoma [137]. Combination regimens of PLK1 inhibitors with chemotherapeutic or targeted agents are actively being explored.

As with *KRAS* mutations, cancers with higher PLK1 expression levels have lower immune activity. Boosting tumor immunity increases the sensitivity of cancer cells to PLK1 inhibition, which may be related to p53 and effects on the cell cycle. BI2536 induced immune cell infiltration and activation. Combining PLK1 inhibition and immunotherapy may therefore be a promising way to achieve better antitumor effects as PLK1 inhibition not only selectively kills cancer cells but also upregulated PD-L1 expression in the remaining cells [111]. A nanoparticle technology called ARAC (Antigen Release Agent and Checkpoint Inhibitor) was developed, combining volasertib with a PD-L1 antibody [138]. It dramatically reduced the effective doses of these drugs and demonstrated responses even in tumors that did not respond to standard immunotherapy [138].

## Discussion

PLK1 is an important oncogene and cell cycle regulator with multiple effects on downstream pathways. It is crucial to CRC progression and is both a prognostic marker and therapeutic target. PLK1 is crucial in the development of drug resistance via effects on p53 pathways, microtubule dynamics, DNA replication, and effects on cancer metabolism. Inhibition of PLK1 expression could resensitize cancer cells and improve response to therapy even in difficult to treat *KRAS* mutant cancers. Multiple small molecule inhibitors of PLK1 are in various stages of development although some have demonstrated a propensity to resistance or excessive toxicity. The logical solution to this resistance seems to be combination therapies to target PLK1 and other pathways. Newer PLK1 inhibitors have better pharmacologic profiles and tolerance.

The recent phase 1b/2 trial of onvansertib with FOLFIRI-bevacizumab in patients with *KRAS* mutant mCRC showed efficacy in second line treatment and demonstrated changes in plasma *KRAS*. Onvansertib and bevacizumab demonstrate synergism in this setting. Evaluation of onvansertib is now moving into the first-line setting along with chemotherapy and bevacizumab with a phase 2 trial of patients with mCRC and *KRAS* or *NRAS* mutation. Just as with traditional chemotherapy, immunotherapy and PLK1 inhibitor combinations may achieve synergistic antitumor efficacy. Combinations with other targeted agents may represent an innovative approach to modulate specific pathways and the tumor microenvironment to improve outcomes.

*RAS* mutations in mCRC are evolving from simply being a negative feature into an important and dynamic target. Despite the failure of many previous attempts, novel RAS-directed agents demonstrate varying levels of response in clinical trials. Combining *KRAS*^*G12C*^ inhibitors with anti-EGFR agents might overcome resistance in mCRC patients, opening the door to using anti-EGFR drugs in a realm previously thought impossible. Similarly, combining *KRAS* blockade with MEKi, PI3K/MTOR inhibitors, or other agents may achieve greater degrees of tumor suppression without rebound activation of other pathways. Combinations with PLK1 inhibitors and ICI may help overcome the immunosuppressive TME associated with *RAS* mutation. Everything from common medications and supplements, like statins and herbs, to novel immunological and hematological interventions, like oncolytic viruses and adoptive t-cell therapies, are being explored to target *KRAS* mutations in CRC. Further clinical investigations are warranted to continue the development of pan-RAS inhibitors and improve outcomes in *RAS* mutant mCRC.
